# Effect of Introduction of Exogenous Strain *Acidithiobacillus thiooxidans* A01 on Structure and Function of Adsorbed and Planktonic Microbial Consortia During Bioleaching of Low-Grade Copper Sulfide

**DOI:** 10.3389/fmicb.2019.03034

**Published:** 2020-01-15

**Authors:** Yi Liu, Junjun Wang, Haijun Hou, Gang Chen, Hongwei Liu, Xueduan Liu, Li Shen

**Affiliations:** ^1^Key Laboratory of Agro-ecological Processes in Subtropical Regions and Taoyuan Station of Agro-ecology Research, Institute of Subtropical Agriculture, Chinese Academy of Sciences, Changsha, China; ^2^School of Minerals Processing and Bioengineering, Central South University, Changsha, China; ^3^Key Laboratory of Biometallurgy, Ministry of Education, Central South University, Changsha, China; ^4^Changsha Folianovo Biotechnology Co. Ltd., Changsha, China

**Keywords:** *Acidithiobacillus thiooxidans* A01, planktonic microbes, adsorbed microbes, functional gene arrays, bioleaching

## Abstract

The introduction of *Acidithiobacillus thiooxidans* A01 strengthens the positive interactions between physiologically distinct microorganisms and enhances the bioleaching ability of the consortium. However, the effect of introducing an exogenous strain, *A. thiooxidans* A01 on the structure and function of the adsorbed and planktonic microbial consortia during bioleaching of low-grade copper sulfide remains unclear. In this study, *A. thiooxidans* A01 was introduced into an indigenous leaching microbial community on the 0th (group B), 24th (group C), and 36th day (group D). Results revealed that the copper leaching efficiency was highest in group D, in which the Cu^2+^ concentration in the solution reached 251.5 mg/L on day 48, which was 18.5% higher than that of the control (group A, no addition of *A. thiooxidans* A01). Restriction fragment length polymorphism (RFLP) analysis of the microbial community in group D revealed the presence of *Leptospirillum ferriphilum*, *Acidithiobacillus ferrooxidans*, *Acidithiobacillus caldus*, *Sulfobacillus* sp., *Acidiphilium* spp., and *Acidithiobacillus albertensis* before introduction of *A. thiooxidans* A01 on the 36th or 48th day; however, *A. albertensis* was absent on day 48 in group A. Further, the proportion of dominant *A. caldus*, *L. ferriphilum*, and *A. ferrooxidans* became altered. The results of real-time PCR in group D showed that *A. thiooxidans* A01 was primarily adsorbed on the surface of the ore, with the adsorption reaching the maxima on day 42; while the free *A. thiooxidans* A01 in solution grew slowly, reaching its maximum concentration on day 45. Compared with that in the control group, the abundance of both free and attached *A. caldus* and *Sulfobacillus* sp. decreased following the introduction of *A. thiooxidans* A01, while that of *L ferriphilum*, *A. ferrooxidans*, and *Acidiphilium* sp. increased. Functional gene arrays data indicated that the abundance of genes involved in sulfide and iron oxidation in *L. ferriphilum* and *A. ferrooxidans*, as well as that of the metal (loid) resistance genes of *A. ferrooxidans*, *L. ferriphilum*, and *Acidiphilium* sp. increased, while the abundance of genes involved in sulfur metabolism in *A. caldus* and *Sulfolobus* spp. decreased. Taken together, these results provide useful information for application of bioleaching of copper sulfide in industry.

## Introduction

Surface water and groundwater are often polluted in the process of mineral exploitation, primarily because sulfide dissolution leads to production of acidic water. These wastewaters often contain high concentration of elements such as copper, zinc, iron, sulfur, and arsenic, and thus called acid mine drainage (AMD). Many extremely eosinophilic microorganisms have been isolated from such acidic environments (Baker and Banfield, 2003; Akcil and Koldas, 2006; Cheng et al., 2009; Marchevsky et al., 2015). Among these microorganisms, different types of interactions such as competition, predation, mutualism, and synergy, have been described (Johnson, 1998). However, many studies have indicated that mixed cultures containing a variety of microorganisms tend to be more robust and more efficient in oxidizing sulfide minerals due to their extensive interactions (Akcil et al., 2007; Zhang et al., 2008; Deng et al., 2017). Further, the ecological function of the microbial community was often associated with its structure and diversity. On the one hand, the unique original microbial compositions may lead to distinct bioleaching efficiencies (Bacelar-Nicolau and Johnson, 1999; Zhang et al., 2007). However, on the other hand, many environmental factors, such as temperature and pH, may result in changes within the community structure and diversity during the bioleaching process (Davis-Belmar et al., 2012), subsequently resulting in varied dissolution rates at different stages (Mukherjee et al., 2004; Demergasso et al., 2005; Johnson, 2008).

In recent years, the continuous development of genome tools has promoted further analysis of microorganisms in complex environments. To study the structure and function of microbial community, functional gene arrays (FGAs), also termed the GeoChip, have been developed and proven to be a useful genomic technology for the thorough investigation of environmental and bioleaching processes (Mukherjea, 1971; Rodrìguez et al., 2003; Schadt et al., 2004; Yin et al., 2007; He et al., 2008, 2011, 2012). In our previous study (Liu et al., 2011b), we artificially constructed multiple co-culture systems of indigenous leaching microbial communities; introduced different types of leaching bacteria in different growth periods of indigenous communities; and studied the introduction mechanism at the gene, cell, and community levels. However, we encountered certain limitations. Firstly, the selected indigenous leaching microbial community was an artificial co-culture system. Generally, natural indigenous leaching microbial communities have a more stable structure and higher diversity (Demergasso et al., 2005; Huang et al., 2018). Secondly, the selected mineral was high purity pyrite not chalcopyrite. Chalcopyrite (CuFeS_2_) accounts for ∼70% of the Earth’s copper and is one of the most wide-spread copper sulfide minerals (Lu et al., 2016). However, the Cu extraction from chalcopyrite through acid leaching is an extremely time-consuming process with low efficiency (Ma et al., 2017). Therefore, it has greater practical significance and value to study introduction mechanisms in the leaching system of low-grade chalcopyrite with indigenous leaching microbial communities.

A leaching system comprises planktonic and adsorbed bacteria (Chen et al., 2017). However, studies on microbial ecology in leaching systems have primarily focused on planktonic microorganisms in leaching solutions, with little knowledge of the microbial ecology of mineral surface adsorption (Klocke et al., 2007; Nkulu et al., 2015). According to previous studies (Sand and Gehrke, 2006; Chen et al., 2008), the metal ion and organic matter content on the mineral surface during the bioleaching process is much higher than that in the leaching solution; indicating that the growth environment of microorganisms on the mineral surface may be quite different from that in the leaching solution. Moreover, the optimal introduction period can differ with the stage of the planktonic microbial community growth period. Therefore, it is of great significance to study the dynamic changes occurring within the community structure and functional genes in the two different growth environments, while also defining the optimal introduction period.

In this study, we selected natural microbial communities as indigenous microbial communities (Liu et al., 2011a). In the low-grade copper sulfide bioleaching system, *A. thiooxidans* A01 was introduced during the stable growth of planktonic and adsorbed microbial communities. Real-time polymerase chain reaction (RT-PCR), restriction fragment length polymorphism (RFLP), and FGAs were used to identify the indigenous populations before and after *A. thiooxidans* A01 introduction. The results underscore the complexity of the bioleaching processes and provide a novel approach to enhance the bioleaching efficiency of an indigenous consortium that may be useful in the bioleaching of low-grade copper sulfide.

## Materials and Methods

### Microorganism Preparation

The microbial consortium used herein has been described in our previous study (Liu et al., 2011b). In brief, the acclimation experiments consisted of an equal mixture of several AMD samples from different copper ores; these experiments were carried out in 0K basal medium containing 0.5% chalcopyrite. The indigenous consortium was obtained in this study after successive subculturing of five generations, with one generation developing every 60 days. *A. thiooxidans* A01 strain (FJ154526), an important member of genus *Acidithiobacillus*, was isolated from the AMD of the Dexing copper mine, China. This organism is an obligate sulfur oxidizer with strong sulfur-oxidizing properties; however, it is unable to utilize ferrous iron as an energy source (Yin et al., 2014). *A. thiooxidans* A01 was also acclimated for five generations in 0K medium containing 0.5% chalcopyrite to adapt the bioleaching system of chalcopyrite before commencing the bioleaching study. The strain was cultivated with elemental sulfur as the sole energy source at pH 2.0, 30°C, and 170 rpm, and subcultured every 2 weeks. The adapted *A. thiooxidans* A01 was used as the exogenous strain in this study. The 0K basal medium (g/L) is composed of (NH_4_)_2_SO_4_ (3), K_2_HPO_4_ (0.5), KCl (0.1), Ca(NO_3_)_2_ (0.01), and MgSO_4_⋅7H_2_O (0.5).

### Mineral Components

The low-grade chalcopyrite used in this bioleaching experiment was obtained from Heilongjiang Province, China. The mineral sample was ground and sieved to obtain a particle size <75 μm. The chemical compositions of copper sulfide ore were analyzed by X-ray fluorescence (XRF), and the result indicated that the contents of Cu, Mo, CaO, and MgO were 0.51, 0.011, 4.63, and 1.29%, respectively. The contents of Ag and Au were 12 and 0.2 g/t, respectively. Mineral phases of copper in low-grade copper sulfide ore were determined by wet chemical analysis according to “DZG20.01-1991” (petromineralogy analysis) (Chun et al., 2016), and the results showed that this copper sulfide presented in the form of primary copper sulfide (74.5%), secondary copper sulfide (23.5%), free copper oxide (1.18%), and combined copper oxide (0.78%). The total content of metallic minerals was 5.71%, and the main metallic minerals included digenite and chalcopyrite, among which the total content of digenite was 0.78% and that of chalcopyrite was 4.85%. From the perspective of gangue minerals, it was mainly quartz, with a total content of 54.2%, including 36.1% alum and 12.5% other mineral elements.

### Bioleaching Experiments

The bioleaching experiments were conducted in 250 mL shake flasks containing 100 mL of autoclaved 0K basal medium with 5% (w/v) ultraviolet-sterilized chalcopyrite sample at 170 rpm for 48 days. The experiment included two parts.

Part I sought to determine the most effective introduction period. The cultivated indigenous consortium and *A. thiooxidans* A01 were, respectively, centrifuged at 10,000 rpm/min for 5 min, and the cell pellet was inoculated into medium at a final cell density of 10^7^ cell/mL. *A. thiooxidans* A01 was introduced into the indigenous consortium on days 0, 24, and 36, respectively (groups B, C, and D). Flasks without the introduction of *A. thiooxidans* A01 were used as a control (group A) and uninoculated flasks were used as abiotic controls. The introduction time was determined according to the 48 days of pre-experiment, in which the concentration of microorganisms adsorbed on the ore surface reached the maximum on the 24th day, while the concentration of microorganisms in solution reached the maximum on the 36th day (data not shown). During the bioleaching experiment, the temperature was adjusted every 8 h, and the circulating temperature was 25, 35, and 45°C, respectively. The circulating temperature was applied since the optimum growth temperature of microorganisms was diverse; hence, varied temperatures may allow the growth of a relatively rich microbial community. In the first 3 days of bioleaching, the pH was daily adjusted to 2.0 using hydrochloric acid and was no longer adjusted once a stable pH was maintained. Samples were withdrawn every 6 days to determine the concentration of copper and ferrous iron in the solution. The evaporated water and sampling loss were supplemented with sterilized distilled water periodically. All experiments were performed in triplicate.

Part II: Based on the experimental results from part I, introduction of *A. thiooxidans* A01 on the 36th day was identified as the most effective introduction period (group D). To analyze the cause for the improved bioleaching efficiency, we determined the microbial community structure, population dynamics, and community functional gene abundance before and after the introduction of *A. thiooxidans* A01 on the 36th day. A total of 39 flasks were inoculated with indigenous consortium (group A), 18 of which were introduced with *A. thiooxidans* A01 on day 36 (group D) as described in part I. The DNA from adsorbed and planktonic bacteria in groups A and D were extracted separately on days 38, 40, 42, 45, and 48. Concurrently, the total microbial DNA (including adsorbed and planktonic microorganisms) was extracted from group A on day 36 (before the introduction of *A. thiooxidans* A01) and from groups A and D on day 48 at the end of the bioleaching process. DNA samples from pure *A. thiooxidans* A01 were also extracted. All experiments were performed in triplicate with three flasks per sampling point. The pH and oxidation-redox potential (ORP) of group A and group D were also determined on days 6, 12, 18, 24, 30, 36, 38, 40, 42, 45, and 48.

### Analytical Techniques

Planktonic bacteria were enumerated by direct counting using a Neubauer chamber hemocytometer. Copper and ferrous iron concentration in solution were determined by inductively coupled plasma-atomic emission spectrometry [ICP-AES: Baird Plasma Spetrovate PS-6 (N+1)] and *o*-phenanthroline spectrophotometric method, respectively. The pH and ORP value were determined using a pH meter (PHSJ-4A, Leici, Shanghai, China) and a platinum electrode with an Ag/AgCl reference electrode (218, Leici, Shanghai, China).

Suspended and adsorbed microorganisms were conducted as described previously (Zeng et al., 2010). The DNA samples from *A. thiooxidans* A01, groups A, B, C, and D were extracted using a TIANamp Bacteria DNA Kit (Tiangen Biotech, Co., Ltd., Beijing, China). The 16S rRNA gene amplification and cloning of consortium A and D on days 36 and 48 were conducted to analyze the microbial community following RFLP analysis. Based on the RFLP profile, a total of 16 representative clones were selected for sequencing. DNA sequence identification was executed using BLAST in GenBank^1^. Microarray hybridization was used to assess population dynamics and functional gene abundance of the microbial community. Real-time quantification polymerase chain reaction (RT-qPCR) was conducted to analyze the population dynamics of *A. thiooxidans* A01. All methods were detailed in our previous study (Liu et al., 2011a). The 16S rRNA sequences were submitted to GenBank, and the accession numbers assigned were JF313219, JF313232-313244, JF429148, and FJ154514.

## Results

### Bioleaching of Low-Grade Copper Sulfide With Consortium A, B, C, and D

**FIGURE 1:**
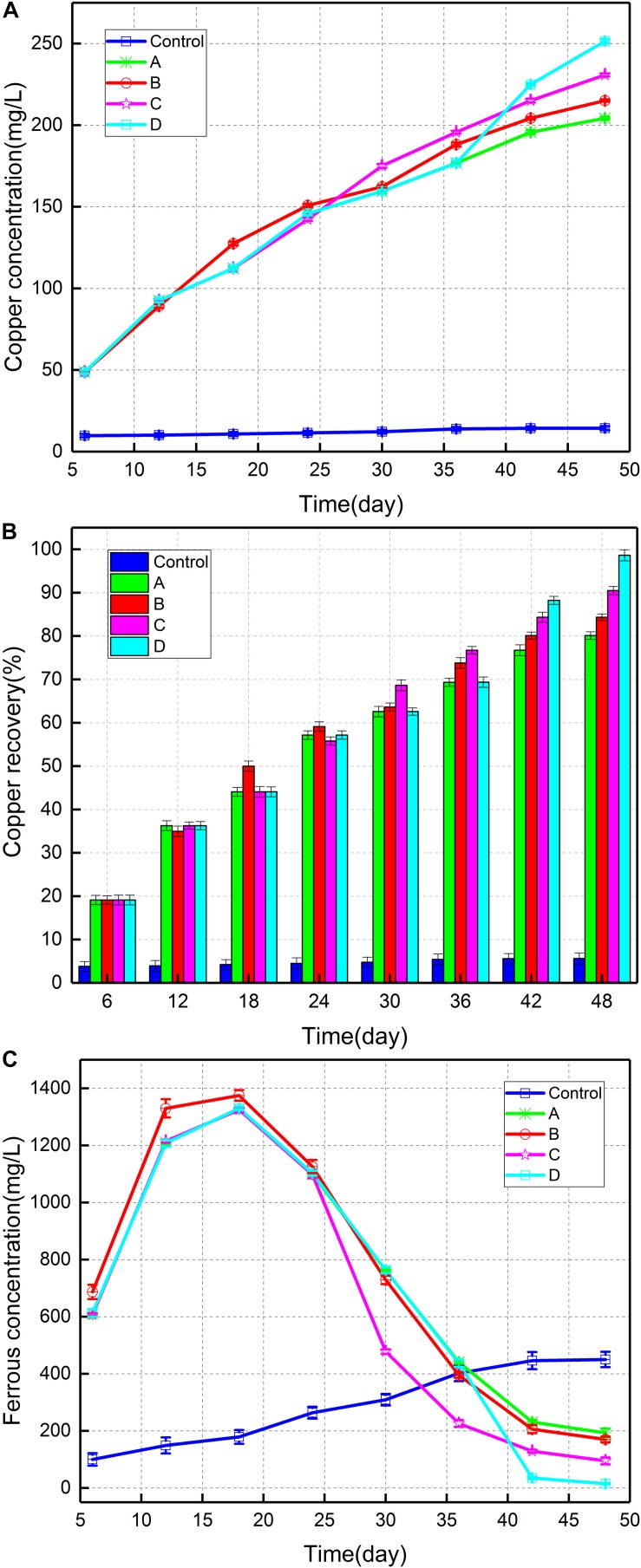
**(A)** Copper concentration of different consortia, **(B)** copper recovery (%), and **(C)** ferrous iron concentration.

Figure 1A depicted the copper concentration in the different groups during the bioleaching experiment. Copper concentration was highest in consortium D (251.5 mg/L) after 48 days, followed by consortium C (230.8 mg/L) and consortium B (215.1 mg/L). Copper content was lowest in group A (204.3 mg/L). Figure 1B showed the copper recovery (%) throughout the entire bioleaching period. The copper recovery was found to gradually increase from 20% on the 6th day to approximately 90% at the end of bioleaching. Following the introduction of *A. thiooxidans* A01 at different growth stages of the indigenous leaching microbial community, copper extraction was observed to improve to varying degrees compared with that in the control, most notably during the stable growth period (up to 98.6%). The copper recovery in groups B, C, and D was 4.3, 10.4, and 18.5% higher than that in group A. Figure 1C displayed the variation in ferrous iron concentration in solution during the bioleaching experiment. The ferrous content of the four groups increased from 629 mg/L on the 6th day to 1,340 mg/L on day 18, after which it began to decrease. The ferrous concentration of group B was slightly higher than other groups in the earlier period and was seen to decline slightly faster than group A in the later period. After introduction of *A. thiooxidans* A01 on day 24, a sudden descent of the ferrous concentration appeared in group C, and the ferrous concentration on day 48 reduced to 95 mg/L. The ferrous concentration in group D decreased after 36 days and finally trended toward zero.

**FIGURE 2:**
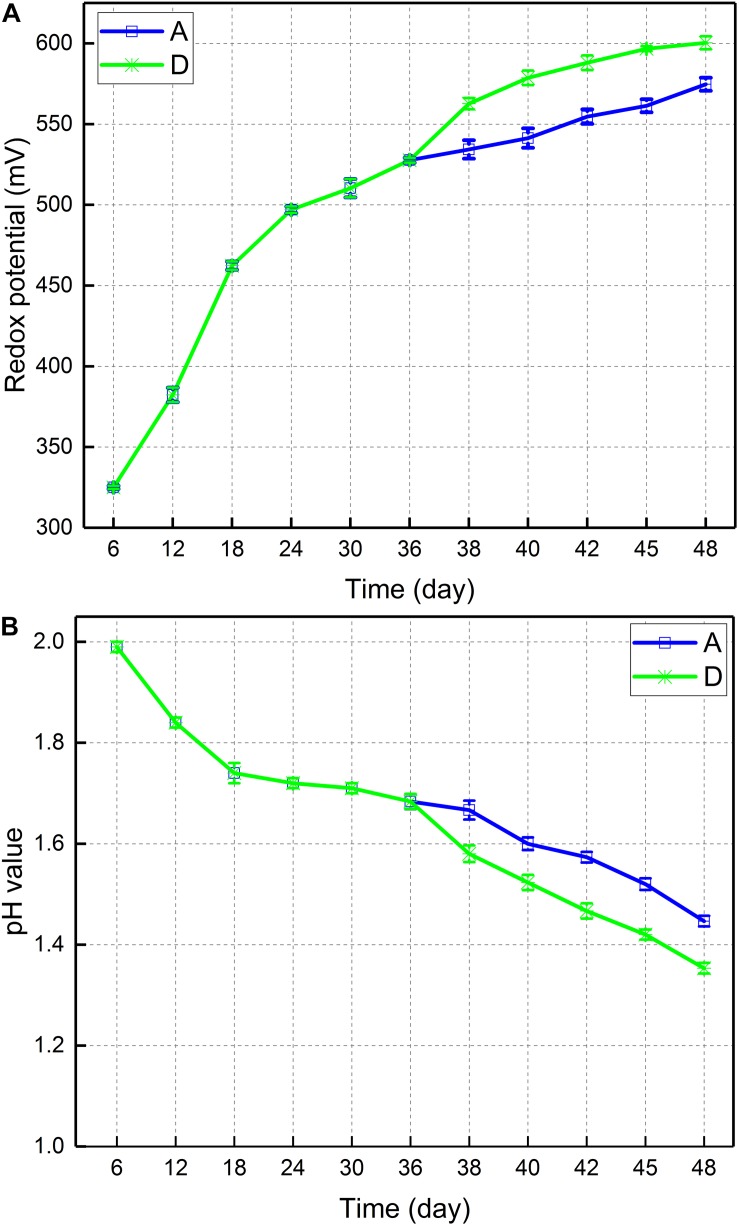
**(A)** Redox potential and **(B)** pH values during bioleaching of chalcopyrite by consortium A and D.

Figure 2A depicted the change in potential of leaching solution of consortium A and D. In consortium A, the potential increased from 534 on day 38 to 575 mV on day 48. The potential of consortium D increased from 563 to 600 mV. Therefore, the introduction of *A. thiooxidans* A01 during the stable growth phase of planktonic bacteria led to a relative increase in potential. Figure 2B displayed the pH change in the leaching solution of consortium A and D. In consortium A, the pH decreased from 1.67 on day 38 to 1.45 on day 48. Similarly, the pH of consortium D decreased from 1.58 to 1.35. Thus, the introduction of *A. thiooxidans* A01 was associated with a relative decrease in pH value. The finding concerning consortium D indicated that the introduction during stable growth of the planktonic bacteria in the native indigenous bioleaching consortium maximally accelerated copper sulfide dissolution.

### Community Structure Analysis

The finding that copper extraction of group D was the highest of the four consortia, prompted further analysis of the effect of introducing *A. thiooxidans* A01 during stable growth (on day 36) on the structure and function of the indigenous consortium, thereby determining why the ecological function of consortium D was improved.

**FIGURE 3:**
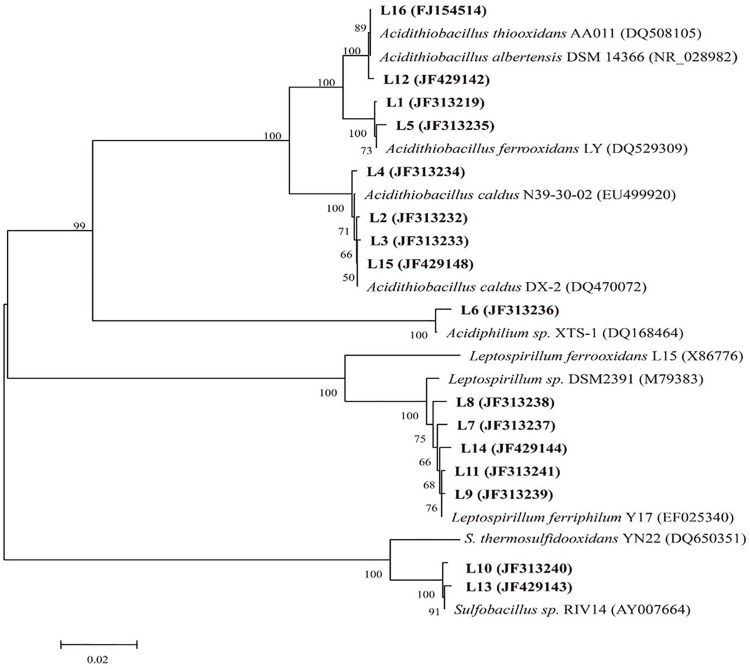
Phylogenetic tree according to the sequences of the partial 16S rRNA gene detected in this study (bold) and the reference sequences downloaded from the NCBI database.

Restriction fragment length polymorphism was applied to analyze the structure of the indigenous consortium A and D. Total DNA was extracted from consortium A on day 36 (before introduction of *A. thiooxidans* A01) and from consortia A and D on day 48 during the bioleaching process. Clones (*n* = 125) of each bacterial 16S rRNA clone library were analyzed by RFLP. A total of 14 operational taxonomic units (OTUs) termed L1 to L16, omitting L4 and L9, were identified (Table 1). The phylogenetic analysis based on all OTUs (including that from the original consortium) was performed to estimate the bacterial population. Six bacterial species including *L. ferriphilum*, *A. ferrooxidans*, *A. caldus*, *Sulfobacillus* sp., *A. albertensis*, and *Acidiphilium* sp. were detected in consortium A (Figure 3). Nearly all were widely common bacteria that were reported in previous studies. Among them, *A. caldus*, *L. ferriphilum*, and *A. ferrooxidans* were the dominant populations in the initial community, and their proportions in consortium A were 38.4, 30.4, and 17.6%, respectively (Table 1). Examination of consortium A on day 48 revealed the same population, except for the absence of *A. albertensis*. The primary difference was that the proportion of each population had changed, and the proportion of *A. caldus*, *L. ferriphilum*, and *A. ferrooxidans* was 43.2, 36, and 10.4%, respectively. Compared with consortium A, consortium D exhibited more diversity at day 48, with six species: *L. ferriphilum* (clones L7, L8, L11, and L14), *A. caldus* (clones L2, L3, and L15), *A. ferrooxidans* (clones L1 and L5), *Sulfobacillus* sp. (clone L10), *A. albertensis* (clone L12), and *Acidiphilium* sp. (clone L6). Alternatively, the proportion of each detected bacteria changed on day 48. The proportions of *A. caldus*, *L. ferriphilum*, and *A. ferrooxidans* were 32, 40, and 20.8%, respectively, in consortium D.

**TABLE 1:** Similarity of 16S rRNA gene sequences from clones arranged into groups according to RFLP patterns to sequences retrieved from databases.

**OTU**	**Closest relative**	**Similarity**	**Frequency (%)**			
	**(accession no.)**	**(%)**	**Control**	**A (day 36)**	**A (day 48)**	**D (day 48)**
L1	*A. ferrooxidans* LY (DQ529309)	99	0	6.4	0	3.2
L2	*A. caldus* DX-2 (DQ470072)	99	31.7	27.2	36	25.6
L3	*A. caldus* N39-30-02 (EU499920)	99	6.4	7.2	5.6	4
L4	*A. caldus* N39-30-02 (EU499920)	99	2.2	0	0	0
L5	*A. ferrooxidans* LY (DQ529309)	100	10.1	11.2	10.4	8.8
L6	*Acidiphilium* sp. XTS-1 (DQ168464)	100	3.6	0.8	0.8	1.6
L7	*L. ferriphilum* Y17 (EF025340)	99	25.2	21.6	26.4	29.6
L8	*L. ferriphilum* Y17 (EF025340)	99	6.4	4.8	5.6	5.6
L9	*L. ferriphilum* Y17 (EF025340)	99	2.9	0	0	0
L10	*Sulfobacillus* sp. RIV14 (AY007664)	99	10.1	5.6	9.6	4.8
L11	*L. ferriphilum* Y17 (EF025340)	99	1.4	2.4	4	2.4
L12	*A. albertensis* DSM 14366	99	0	4.8	0	3.2
L13	*Sulfobacillus* sp. RIV14 (AY007664)	99	0	2.4	0	0
L14	*L. ferriphilum* Y17 (EF025340)	99	0	1.6	0	2.4
L15	*A. caldus* N39-30-02 (EU499920)	99	0	4	1.6	0
L16	*A. thiooxidans* AA011 (DQ508105)	100	0	0	0	8.8

### *A. thiooxidans* A01 Dynamics Analysis

**FIGURE 4:**
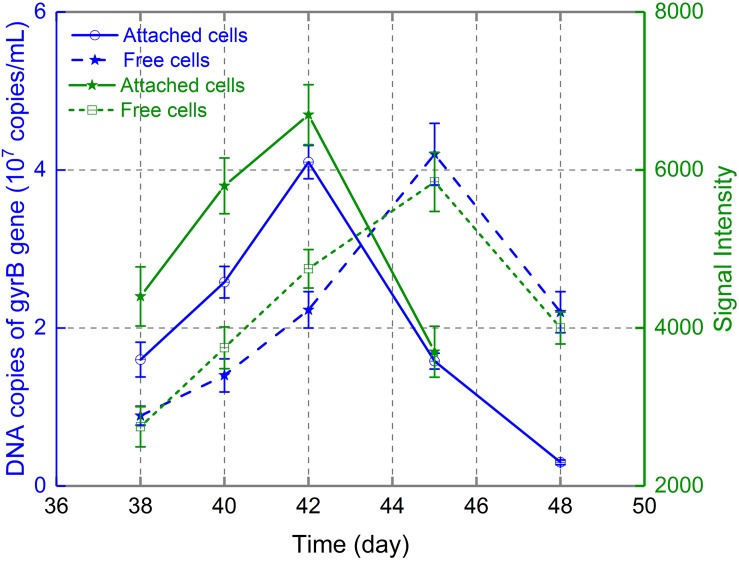
Dynamic change of *A. thiooxidans* A01 in consortium D.

As the ratio of 16S rRNA copies to cell number is constant (Demergasso et al., 2005), the variation in 16S rRNA copy number in real-time PCR can reflect the variation in cell number during various bioleaching processes (Zhang X. et al., 2015; Deng et al., 2017). Therefore, real-time PCR was used to monitor the dynamic changes in free and attached *A. thiooxidans* A01 in this study. Extracted DNA from free and attached cells at days 38, 40, 42, 45, and 48 during the bioleaching of low-grade chalcopyrite was used for standard and RT-PCR. Figure 4 depicted the changes in 16S rRNA copies of *A. thiooxidans* A01 in leaching solution and on the mineral surface after its introduction to the indigenous consortium. The abundance of *A. thiooxidans* A01 attached to the mineral surface reached maximum density (4.08 × 10^7^ copies/mL) at day 42 and decreased to 3.03 × 10^6^ copies/mL by day 48. Planktonic *A. thiooxidans* A01 increased gradually from 7.82 × 10^6^ copies/mL at day 38 to 4.32 × 10^7^ copies/mL at day 45, and then decreased to 2.12 × 10^7^ copies/mL at day 48. The microarray hybridization data showed a similar pattern.

### Dominant Population Dynamics Analysis

**FIGURE 5:**
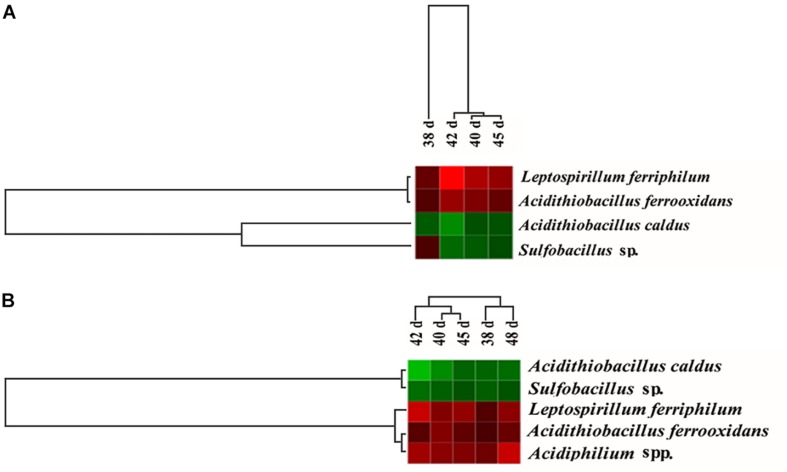
Hierarchical cluster analysis of the dynamics of the attached microorganisms **(A)** and the planktonic microorganisms **(B)** from 38 to 45 days according to the hybridization signal intensity ratios compared with the original consortium. Red means an obvious increase in hybridization signal, black means no significant change, and green means an obvious decrease in hybridization signal.

The dominant population dynamics of the free and attached microorganisms was monitored using FGAs constructed as previously detailed and containing all acquired 16S rRNA sequences of acidophiles from GenBank (Yin et al., 2007; He et al., 2008). Thus, the array was suitable to analyze microbial structures and dynamics during the bioleaching processes. Figure 5 depicted the variation in the 16S rRNA hybridization signal ratios of the dominant population derived from the planktonic and attached bacteria in the consortium A and D. The abundance of the attached *L. ferriphilum* and *A. ferrooxidans* increased in consortium D compared with that in consortium A, while that of *A. caldus* and *Sulfobacillus* sp. was reduced (Figure 5A). The abundance of planktonic *L. ferriphilum*, *A. ferrooxidans*, and *Acidiphilium* spp. increased following introduction of *A. thiooxidans* A01, while the abundance of attached *A. caldus* and *Sulfobacillus* sp. decreased (Figure 5B).

### Changes in Microbial Functional Genes

**FIGURE 6:**
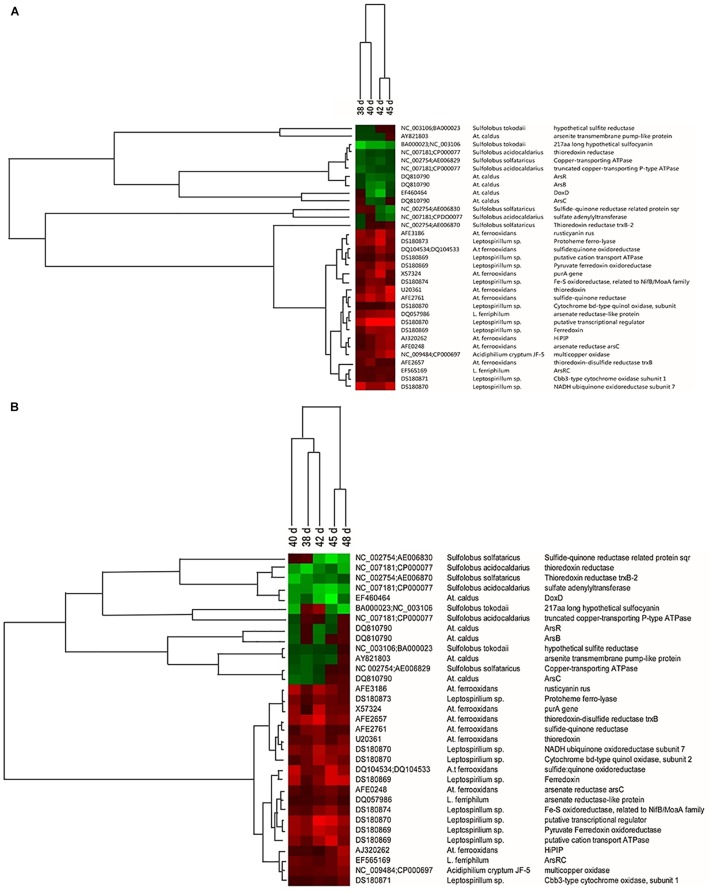
Hierarchical cluster analysis of the functional gene changes in ore surface **(A)** and leaching solution **(B)** after introduction of *A. thiooxidans* A01 from 38 to 45 days according to the hybridization signal intensity ratios compared with the control consortium. Red means an obvious increase in hybridization signal, black means no significant change, and green means an obvious decrease in hybridization signal.

To verify how the introduction of *A. thiooxidans* A01 influenced microbial ecological functions, the changes in functional genes of the planktonic and attached microorganisms were analyzed in detail. The consortium from group A was used as a control. Key genes involved in sulfur metabolism, iron oxidation, and metal resistance with the high hybridization signal intensities were clustered (Figure 6). With respect to functional genes related to iron metabolism, the abundance of genes encoding protoheme ferrolyase from *L. ferriphilum* increased significantly in both attached and planktonic samples. For functional genes related to sulfur metabolism, the abundance of the genes encoding sulfide-quinone reductase from *A. ferrooxidans* was obviously increased. However, the abundance of genes involved in sulfur metabolism from *A. caldus* and *Sulfolobus* spp. was decreased during the leaching process. For example, the abundance of the *doxD* gene related to the oxidation of inorganic sulfur complex from *A. caldus* decreased obviously in attached and planktonic samples. Furthermore, the abundance of some genes involved in metal resistance from *A. ferrooxidans*, *L. ferriphilum*, and *Acidiphilium* sp. was increased in consortium D.

## Discussion

The introduction of *A. thiooxidans* A01 was advantageous for the dissolution of chalcopyrite (Figures 1A,B). Compared with that in the control group (group A), the redox potential increased immediately after *A. thiooxidans* A01 introduction (500–600 mv), which increased the oxidation of chalcopyrite and copper concentration. In addition, the pH of the solution decreased significantly with the subsequent rapid increase of redox potential (Figure 2A). This was consistent with the results of previous studies, wherein the redox potential of the solution was related to the ratio of Fe^3+^/Fe^2+^ (Zhang et al., 2014; Huang et al., 2018). The reason may be that the concentration of Fe^3+^ in the solution increased and the oxidation of sulfur produced a relatively large amount of sulfuric acid (Zhang X. et al., 2015). The results of the ferrous concentration in this study confirmed this finding (Figure 1C), as for which the ferrous concentration rapidly decreased following the introduction of *A. thiooxidans* A01, regardless of when it was introduced, and the rapid decrease of ferrous iron was attributed to the rapid oxidation of ferric iron. In accordance with the Nernst equation (Romero et al., 2003), the Fe^3+^/Fe^2+^ ratio is positively related to the redox potential. The pH value showed an overall downward trend during the entire bioleaching process. This explains the relatively high sulfur oxidation and precipitation of iron (jarosite) that have been previously described (Sandström and Petersson, 1997; Gómez et al., 1999; Zhang L. et al., 2015). These physicochemical parameter results were similar to those reported by Ma et al. (2017) in which they investigated the effect of an artificially designed proportion of microorganisms on chalcopyrite bioleaching, and found that the group with more sulfur-oxidizing bacteria induced higher copper extraction and redox potential as well as a lower pH and a faster oxidation of ferrous iron. Together, these results indicate that the introduction of sulfur-oxidizing bacteria can effectively promote the bioleaching of chalcopyrite.

Compared with the composition and structure of consortium A at day 48, the proportion of various microorganisms changed in consortium A and D at day 48 (Table 1). The results showed that the introduction of *A. thiooxidans* A01 during the stable period could promote the growth of *L. ferriphilum* and inhibit the growth of *A. caldus*. Similarly, the introduction of *A. thiooxidans* A01 during the stable period had differing effects on each population in consortium D (Figure 5). The reason for this observation may be that *A. thiooxidans* A01, as an obligate sulfur-oxidizing bacterium, was bound to join the competition of sulfur energy due to the need for growth after its introduction into the indigenous community. As a result of the intensified competition, the growth of identical sulfur-oxidizing bacteria, *A. caldus* and *Sulfobacillus* sp., were also inevitably affected, leading to their growth inhibition. For iron-oxidizing bacteria that use Fe^2+^ as an energy source, the introduction of *A. thiooxidans* A01 accelerated the oxidative decomposition of minerals (the direct mechanism), which led to the production of more Fe^2+^ (Liu et al., 2006). *L. ferriphilum* and *A. ferrooxidans*, thus, obtained increased energy to grow, thereby increasing their abundance in the community (Ma et al., 2018). Moreover, the introduction of exotic species could stimulate the enhancement of the metabolic activity of the community and thus provided more carbon and energy sources for the growth of heterotrophic bacteria, which may be one of the reasons for the increased abundance of heterotrophic *Acidiphilium* sp. in the community (Ullrich et al., 2015). In addition, in the latter stage, the abundance of organic matter accumulated from microbial biomass and metabolites provided more energy for the growth of heterotrophic bacteria. *Acidiphilium* sp., as a heterotrophic acidophile, may play an important role in the consumption of organics and, thus, reduce the inhibitory effect of organics on *A. ferrooxidans* and *L. ferriphilum* (Huang et al., 2018). For the planktonic microbial population in the leaching solution, the trend was consistent with that of the attached population following introduction. However, the degree of change varied for different timepoint or populations. According to previous studies (Harneit et al., 2006; Africa et al., 2012), microorganisms from mineral adapted cultures are capable of attaching to minerals more efficiently, and produce increased levels of EPS, which may concentrate the ferric iron to oxidatively attack chalcopyrite. When ferrous iron becomes trapped in the EPS layer, it gets released into the solution, thereby allowing the microorganisms in solution to grow rapidly (Zeng et al., 2010). Moreover, in the later stages of bioleaching, large amounts of jarosite and sulfur film form on the mineral surface, effectively blocking further oxidation. Thus, the introduction of *A. thiooxidans* A01 at this time period would attach to the mineral surface, thereby partially eliminating the inhibition of sulfur, which ultimately results in higher copper efficiency (Dong et al., 2013; Ma et al., 2017).

The changes in community function genes before and after introduction were also analyzed (Figure 6). With respect to functional genes related to sulfide oxidation, thioredoxin, sulfide: quinone oxidoreductase, sulfide–quinone reductase, thioredoxin–disulfide reductase *trxB*, and *HiPIP* were observed in *A. ferrooxidans* (Wakai et al., 2004). The abundance of these genes was significantly increased in both attached and planktonic samples, while the abundance of genes involved in sulfur metabolism from *A. caldus* and *Sulfolobus* spp. such as thioredoxin reductase, *DoxD*, and sulfide-quinone reductase*-*related protein *sqr* was decreased. However, in a previous study (Liu et al., 2011a), the abundance of genes involved in sulfur metabolism from *A. caldus* and *Sulfolobus* spp. was increased with increasing number of subculture, while genes involved in sulfur metabolism from *A. ferrooxidans* were decreased. These opposing results indicate that the introduction of *A. thiooxidans* A01 may exhibit synergistic effects with those of *A. ferrooxidans* and *L. ferriphilum*; however, it competes for energy with *Sulfolobus* spp. and *A. caldus*. Further, iron metabolism-related genes such as rusticyanin (*rus*) from *A. ferrooxidans*, ferredoxin and protoheme ferro-lyase from *L. ferriphilum*, and electron transfer chain-related genes were all increased following introduction of *A. thiooxidans* A01, thereby enhancing ferrous oxidation activity (Liu W. et al., 2011). Additionally, the abundance of arsenate reductase (*arsC*), arsenate reductase-like protein, and *ArsRC* genes involved in metal (loid) resistance in *A. ferrooxidans*, *L. ferriphilum*, and *Acidiphilium* sp. was increased in group D. Arsenic is often associated with sulfide minerals, and its toxicity becomes apparent after the dissolution of the mineral (Drewniak and Sklodowska, 2013). It has been reported that the *arsC* gene is responsible for reducing arsenate to arsenite, and that the *arsB* gene is involved in arsenite efflux, while *arsR* may encode a repressor of *arsB* (Butcher et al., 2000). The introduction of *A. thiooxidans* A01 promoted the further dissolution of chalcopyrite, resulting in more arsenic being released into the solution. These upregulated genes helped them to resist pressure and ensured survival (Luo et al., 2009).

## Conclusion

The exogenous species *A. thiooxidans* A01 was introduced into an indigenous consortium on days 0, 24, and 36 (bioleaching cycle spanned 48 days). Copper concentration was 215.1, 230.8, and 251.5 mg/L, respectively, in the bioleaching system. After the introduction of *A. thiooxidans* A01, *L. ferriphilum*, *A. ferrooxidans*, *A. caldus*, *Sulfobacillus* sp., *A. albertensis*, and *Acidiphilium* were continuously detected, although their proportions changed during bioleaching. Only five microbial species (except for *A. albertensis*) were detected, and the proportions of *A. caldus*, *L. ferriphilum*, and *A. ferrooxidans* were 43.2, 36, and 10.45% in the control group. Real-time PCR and FGAs data suggested that *A. thiooxidans* A01 introduced at day 36 primarily attached to the mineral surface. The absorbance peaked at day 42 and then declined. In the supernatant, the cell number of *A. thiooxidans* A01 was initially low, peaked at day 45, and then decreased. The abundance of both attached and planktonic *A. caldus* and *Sulfobacillus* sp. decreased after the introduction of *A. thiooxidans* A01, while the abundance of *L. ferriphilum*, *A. ferrooxidans*, and *Acidiphilium* sp. all increased. FGAs data indicated that the abundance of genes involved in sulfide and iron oxidation from *L. ferriphilum* and *A. ferrooxidans*, and the metal (loid) resistance gene from *A. ferrooxidans*, *L. ferriphilum*, and *Acidiphilium* sp. increased, while the genes involved in sulfur metabolism from *A. caldus* and *Sulfolobus* spp. decreased. This research is of great significance to study the introduction mechanism of indigenous leaching microbial communities.

## Data Availability Statement

All datasets generated for this study are included in the article/supplementary material.

## Author Contributions

YL designed and performed the experiments. YL and JW wrote the manuscript. HH and GC assisted in performing some experiments. XL, HL, and LS revised the manuscript. All authors read and approved the submitted version of the manuscript.

## Conflict of Interest

GC was employed by company Changsha Folianovo Biotechnology Ltd. The remaining authors declare that the research was conducted in the absence of any commercial or financial relationships that could be construed as a potential conflict of interest.

## Footnotes

1 http://www.ncbi.nlm.nih.gov/BLAST/
